# Assessment of Wnt pathway selected gene expression levels in peripheral blood mononuclear cells (PBMCs) of postmenopausal patients with low bone mass

**DOI:** 10.17305/bjbms.2020.5179

**Published:** 2021-08

**Authors:** Michal Stuss, Monika Migdalska-Sek, Ewa Brzezianska-Lasota, Marta Michalska-Kasiczak, Pawel Bazela, Ewa Sewerynek

**Affiliations:** 1 Department of Endocrine Disorders and Bone Metabolism, Chair of Endocrinology, Medical University of Lodz, Lodz, Poland; 2 Outpatient Clinic of Osteoporosis, Regional Center of Menopause and Osteoporosis, Military Medical Academy Memorial Teaching Hospital of the Medical University of Lodz – Central Veterans’ Hospital, Lodz, Poland; 3 Department of Biomedicine and Genetics, Chair of Biology and Medical Parasitology, Medical University of Lodz, Lodz, Poland

**Keywords:** Osteoporosis, Wnt pathway, PBMC, expression, BMD, fractures

## Abstract

The purpose of the study was to assess the expression of selected genes of the Wnt pathway: *APC, AXIN1, CTNNB1, DKK1, GSK3B, KREMEN1, SFRP1*, and *WNT1* in peripheral blood mononuclear cells (PBMC) of patients, selected in consideration of their bone mineral density (BMD), and the occurrence of low-energy fractures. The study involved 45 postmenopausal women, divided into four groups, according to BMD and fracture history. Measurements of laboratory parameters and RNA expression in PBMC cells were carried out in material, collected once at the inclusion visit. The densitometric examination was performed on all participants. In the analysis of the relative expression levels (RELs) of the studied genes in the entire population, we observed an overexpression for *SFRP1* in 100% of samples and *WNT1*. In addition, the REL of *DKK1, APC*, and *GSK3B* genes were slightly elevated versus the calibrator. In contrast, *CTNNB1* and *AXIN1* presented with a slightly decreased RELs. Analysis did not show any significant differences among the groups in the relative gene expression levels (*p* < 0.05) of particular genes. However, we have observed quite numerous interesting correlations between the expression of the studied genes and BMD, the presence of fractures, and laboratory parameters, both in the whole studied population as well as in selected groups. In conclusion, the high level of *CTNNB1* expression maintains normal BMD and/or protects against fractures. It also appears that the changes in expression levels of the Wnt pathway genes in PBMCs reflect the expected changes in bone tissue.

## INTRODUCTION

The Wnt pathway is crucial for bone reconstruction ­process and plays a significant role in a number of important processes, including, among others, the expression control of other genes, the control of maturation, differentiation, apoptosis and adhesion of many types of cells [1-4].

Two Wnt pathway types are distinguished: β-catenin-dependent canonical pathway: Classical and non-classical. In case of the classical pathway, the binding of one of the pathway ligands (e.g., Wnt1) to the Fzd-LRP5/6 (Frizzled - Low-density lipoprotein Receptor-related Protein 5 or 6) complex induces recruitment and activation of dishevelled (Dvl) intracellular protein and its binding to the cytoplasmic part of the Fzd protein. In consequence, β-catenin phosphorylation and its degradation in proteasomes are inhibited by *GSK3B*. Dephosphorylated (activated and stable) β-catenin undergoes translocation to the cell nucleus, where it binds with T-cell transcription factor/lymphocyte enhancer factor-1 (TCF/LEF-1), initiating a transcription of the pathway-controlled genes, associated with the other processes of osteoblast differentiation and maturation [1]. A formation of the heterodimeric β-catenin/TCF/LEF complex is of key importance to trigger the transcription of target genes and their expression. The activation of the canonical Wnt pathway also indirectly contributes to the suppression of bone resorption process via effects on osteoclastogenesis [5-7]. The above mentioned APC, Axin, β-catenin, and *GSK3B* proteins are, respectively, encoded by the *APC* (*locus* 5q22.2)*, AXIN1* (*locus* 16p13.3)*, CTNNB1* (*locus* 3p22.1), and *GSK3B* (*locus* 3q13.33) genes. The Wnt1 (Wingless-Type MMTV Integration Site Family, Member 1; the Wnt Family Member 1) encoded by *WNT1* (*locus* 12q13.12) is one of the canonical Wnt signaling pathway activating ligands. It has been demonstrated that mutations, which take place within this gene, may lead to early onset severe osteoporosis or to osteogenesis imperfecta [8,9]. Wnt pathway has also its inhibitors. Dkk1 protein encoded by *DKK1* gene (*locus* 10q21.1) is one of the Dickkopf family proteins and it is primarily found in osteocytes and osteoblasts [10]. Dkk1 inhibits the Wnt pathway by binding with LRP5 or 6 [11], fairly competitive to other ligands, while its antagonistic function is substantially strengthened by the presence of Kremen proteins [12]. Kremen1 (Krm1) and Kremen2 (Krm2), encoded by *KREMEN1* (*locus* 22q12.1) and *KREMEN2* (*locus* 16p13.3), respectively, are high affinity receptors for Dkk1, which functionally cooperate with this protein, synergistically blocking the canonical Wnt pathway [13]. The soluble Fzd-related proteins (sFRP) are the largest family of Wnt pathway inhibitors, their structure being similar to that of the Fzd protein [14]. The *SFRP1* (*locus* 8p11.21) encodes sFRP1 protein, which is one of the most important sFRP members.

There is evidence showing that morphological-functional changes in peripheral blood mononuclear cells (PBMC) are likely to reflect the severity of osteoporosis in postmenopausal women [15-20]. PBMC are also easily accessible and relatively non-invasive research material, and potentially their activity may also reflect changes in bone cells, including the canonical Wnt pathway [20].

The aim of our study was to assess the expression of the following Wnt pathway genes*: APC, AXIN1, CTNNB1, DKK1, GSK3B, KREMEN1, SFRP1*, and *WNT1* in PBMC of patients, selected in consideration of their bone mineral density (BMD) and the occurrence of low-energy fractures. We also wanted to evaluate the presence of possible correlations between the mRNA expression levels of the above-mentioned Wnt pathway genes and BMD or previous fractures. Ultimately, we intended to verify whether the chosen research model could have experimental or clinical application in assessment of Wnt pathway gene expression. Our study is the first attempt to assess differences in the Wnt pathway gene expression in PBMC, in the aspect of postmenopausal osteoporosis and osteoporotic fractures.

## MATERIALS AND METHODS

The study involved 45 postmenopausal women at the age of 50-82 years (66.13 ± 8.26 mean ± standard deviation [SD]), all of them being patients the Regional Menopause and Osteoporosis Centre of the Military Medical Academy Memorial Teaching Hospital of the Medical University of Lodz during the years of 2015-2016. Each patient was recommended a daily intake of 800-2000 IU vitamin D3 plus a calcium preparation in a total amount ensuring its daily supply of approximately 1500 mg (elemental calcium), taking into account daily nutritional habits.

The exclusion criteria included primarily: Chronic intake of pharmacological agents affecting the bone turnover, the presence of chronic systemic diseases which may significantly interfere with bone metabolism, vitamin D deficit (the concentration of 25(OH)D or of vitamin D total <20 ng/ml), malabsorption, and no written consent to participate in the study.

The population was divided into the following 4 groups, depending on BMD and previous osteoporotic fractures:

1. Patients with osteopenia but without fractures; n=10 (Group 1)
2. Patients with osteopenia and with low energy fractures; n=13 (Group 2)
3. Patients with densitometric osteoporosis (T score ≤ −2.5 SD) without fractures; n=13 (Group 3)
4. Patients with densitometric osteoporosis (T score ≤−2.5 SD) with low energy fractures; n=9 (Group 4). See Supplementary Table 1 for baseline characteristics of the study patients.

Following the WHO’s guidelines, the densitometric features of osteoporosis were defined as BMD of the femoral neck and/or of the total hip (TH) and/or of the lumbar spine (LS) ≤−2.5 SD. Osteopenia was diagnosed in those patients, whose BMD, measured in the above-mentioned localizations, achieved T score ≤−1.0 SD and >−2.5 SD.

### Blood collection procedure

Nine milliliters of full blood were collected on EDTA from each of the patients to isolate the genetic material (RNA). Blood samples (5 ml) were also taken on clot for the determination of total calcium, phosphates, vitamin D, parathormone, and other laboratory tests necessary to exclude ­secondary causes of low bone mass and/or of fractures. In addition, the patients were obligated to provide a representative sample from 24-hour urine collection in order to determine the excretion of calcium and phosphate with urine. In each patient ­densitometry of the hip and lumbar spine as well as VFA (vertebral fracture assessment) were performed.

### Densitometry

BMD evaluation was carried out by the dual-energy X-ray absorptiometry (DEXA) technique, using a GE Lunar Prodigy device. The lowest approved accuracy level for technicians at our Centre does not go beyond the following values: 1.9% (LSC = 5.3%) for the LS, 1.8% (LSC = 5.0%) for the TH, and 2% (LSC = 5.5%) for the femoral neck.

### Ethical statement

The study was approved by the local bioethics committee of the Medical University of Lodz, No. RNN/136/15/KE. Each patient signed an informed consent, before participating in the study.

We analyzed the relative expression level of the following genes: *APC, AXIN1, CTNNB1, DKK1, GSK3B, KREMEN1, SFRP1*, and *WNT1*. The collected blood (EDTA) was centrifuged in density gradient, using the Histopaque-1077 agent (Sigma-Aldrich, Poznań, Polska) according to manufacturer’s protocol.

A total RNA isolation from lymphocytes was done, using a mirVana™ miRNA Isolation Kit with a phenol:chloroform mixture (Life Technologies, Carlsbad, CA), following the manufacturer’s recommendations. The quality and quantity of isolated RNA were spectrophotometrically assessed by measuring absorbance at the wave length of 260/280 nm (BioPhotometer™ Plus, Eppendorf, Hamburg, Germany). RNA with the 260/280 nm coefficient values within the range of 1.8-2.0 was regarded to be of high quality and was then used for complementary DNA (cDNA) synthesis.

A cDNA was transcribed from 100 ng of total RNA, using a High-Capacity cDNA Reverse Transcription Kit (Applied Biosystems, Carlsbad, CA) in a total volume of 20 ml per reaction. The reaction mixture contained (RT): 10× RT buffer, 25× of dNTP mixture (100 mM), 10× RT of the starters, MultiScribe™ reverse transcriptase, the RNase inhibitor, and nuclease-free water. The RT reaction was carried out in a SureCycler 8800, Agilent Technologies, Santa Clara, CA, using the following conditions: 10 minutes in 25°C, 120 minutes in 37°C, and then the samples were heated up to 85°C for 5 minutes and maintained in the temperature of 4°C.

An analysis of the relative expression level (qPCR) of selected genes was carried out in an Applied Biosystems 7900HT fast real-time PCR System device (Applied Biosystems, Carlsbad, CA) for 39 cycles in temperature of 60°C, in triple repetitions for each sample. The qPCR evaluation was performed, using TaqMan probes for the following studied genes: *APC* (Hs01568269_m1), *AXIN1* (Hs00394718_m1), *CTNNB1* (Hs00355049_m1), *DKK1* (Hs00183740_m1), *GSK3B* (Hs01047719_m1), *KREMEN1* (Hs00980701_m1), *SFRP1* (Hs00610060_m1), *WNT1* (Hs01011247_m1), and *GAPDH* (Hs99999905_m1) as a reference gene. The PCR mixture contained: cDNA (1-100 ng), 20× TaqMan^®^ Gene Expression Assay, 2× KAPA PROBE FAST ABI Prism^®^ qPCR Kit (Kapa Biosystems Ltd., London, UK), and RNase-free water in a total volume of 20 μl. The expression level (RQ value) of the studied genes was calculated by the ΔΔCT method (TaqMan Relative Quantification Assay software, Applied Biosystems, Carlsbad, CA) with adaptation to the expression level of the *GAPDH* endogenous control and with reference to the expression level of the calibrator (RNA isolated from the separation of lymphocytes from a healthy postmenopausal patients), the RQ value for which was equal to 1.

### Statistical analysis

The experimental data are presented as means ± SD ± confidence interval (SEM×1.96). Since distributions of most of the variables were significantly different from a normal distribution (Shapiro-Wilk test), we used the non-parametric tests: The Mann–Whitney U test was used for two-group comparisons, or the Kruskal–Wallis test for multiple group comparisons. The Spearman rank correlation coefficient was used to measure the direction and strength of the association for individual variables. A statistical analysis was carried out by means of the Statistica 13.1 software package (StatSoft, Cracow, Poland). For all the analyses, *p* < 0.05 was accepted as the level of significance.

## RESULTS

### Analysis of the relative expression levels of the studied genes in the entire population

In the analysis of the relative expression levels (REL) of the studied genes in the entire population, the highest expression level (RQ >1) was observed for the *SFRP1* in 100% of the studied samples (the mean RQ=49.95), while the lowest (RQ<1) was found for the *CTNNB1* in 32% of the studied samples (the mean RQ=0.894) versus the calibrator. A distinct overexpression of the *WNT1* (the mean RQ=11.54) and a decreased expression of the *AXIN1* (the mean RQ=0.95) were also observed. The REL of *DKK1, APC*, and *GSK3B* were slightly elevated (the mean RQ being 1.43, 1.31, and 1.10, respectively), while the REL of the *KREMEN1* was approximated (the mean RQ=1.04) versus the calibrator. The obtained values are presented in Table 1.

**TABLE 1:**
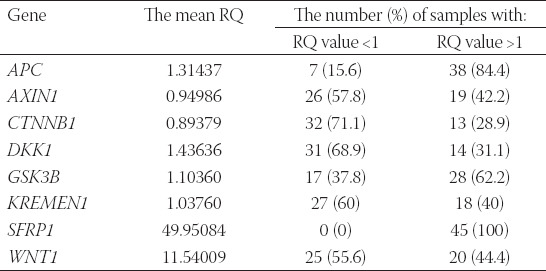
The mean RQ value of the studied genes in the whole study population and the percentage of samples with reduced/increased expression levels relative to the calibrator

### Analysis of the relative expression levels of the studied genes in the particular groups of patients

**FIGURE 1:**
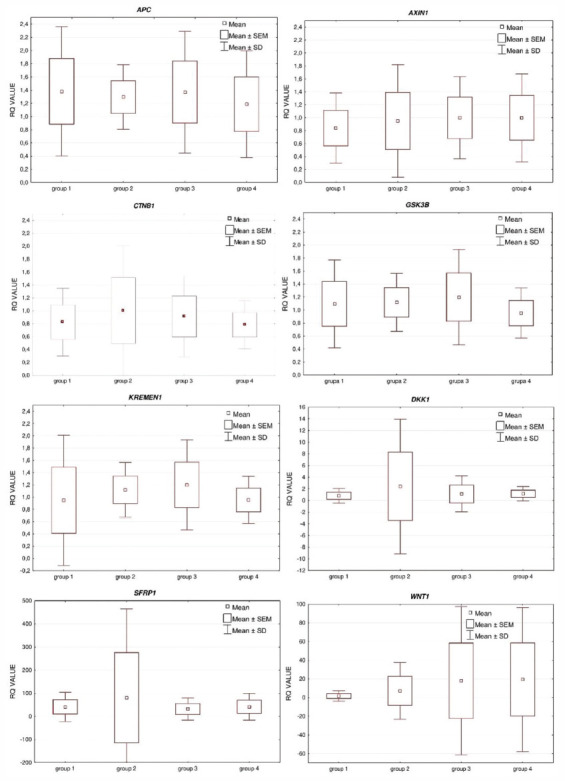
The expression levels of the studied genes (mean RQ ± SD values) in the whole study population, taking into account its division into subgroups.

REL of the studied genes were compared in particular groups of patients. The following differences were observed in RQ for the studied genes: *APC, AXIN1, CTNNB1, DKK1, GSK3B, KREMEN1, SFRP1*, and *WNT1* in particular groups. A statistical analysis did not show any significant differences among the study groups (1-4) in the REL (RQ) (*p* > 0.05) of particular genes (Figure 1).

### Analysis of the concentrations of total calcium, phosphates in serum and urine from 24 hours, alkaline phosphatase in serum, parathormone, and vitamin D (the data are not shown)

The concentrations of total calcium, phosphates in serum and urine from 24 hours, alkaline phosphatase in serum and PTH, and 25(OH)D did not show any statistically significant differences among the study groups (*p* > 0.05).

### Correlations between the expression levels of the studied genes and the age of patients, bone mineral density, fracture history, and results of laboratory tests

A statistical analysis showed some correlations among the RELs of some of the studied genes, both in the entire study population and in particular groups (Supplementary Table 2).

### Gene expression versus age

Regarding the group of patients with advanced osteoporosis (Group 4), the expression of *CTNNB1* decreased inversely proportional to the age of the patients (R=−0.736, *p* = 0.0236). The studied groups were not significantly different with regards to the age of patients (*p* > 0.05).

### Gene expression versus bone mineral density

In the whole studied population, significant positive correlations were identified between the REL of *CTNNB1* and TH T-score (R=0.309, *p* = 0.039) and total hip (TH) BMD (R=0.305, *p* = 0.042). There was also a tendency toward negative correlations (R=−0.262, *p* = 0.082 and R=−0.236, *p* = 0.118) between TH T-score and BMD and the REL of *DKK1* gene. In addition, there were significant positive correlations between the expression of *KREMEN1* and LS BMD and T-score (R=0.338, *p* = 0.025 and R=0.347, *p* = 0.021, respectively).

In the group of patients with osteopenia and without fractures (Group 1), the expression of *KREMEN1* (R=−0.672, *p* = 0.033) negatively correlated with TH T-score and BMD. We also observed a tendency toward a negative correlation in case of TH T-score (R=−0.596, *p* = 0.069). Analogous correlations were also found in case of *SFRP1*, the REL of which negatively correlated with TH T-score (R=−0.711, *p* = 0.021) and TH BMD (R=−0.745, *p* = 0.014).

In the subpopulation of patients with osteopenia and fractures (Group 2), there was tendency to a positive correlation between the REL of *CTNNB1* and TH BMD (R=0.533; *p* = 0.061) and TH T-score (R=0.522, *p* = 0.067).

In Group 3 (postmenopausal osteoporosis, without fractures), the REL of: *GSK3B* and *SFRP1* positively correlated with TH BMD (R=0.560, *p* = 0.046 and R=0.703, *p* = 0.007) and TH T-score (R=0.560, *p* = 0.046 and R=0.703, *p* = 0.007), respectively.

Regarding the patients with advanced osteoporosis (Group 4), we found out negative correlations between LS T-score and the REL of: *GSK3B* (R=−0.810, *p* = 0.008). In addition, a tendency was observed toward negative correlation between the expression of *DKK1* and TH T-score (R=−0.636, *p* = 0.065) and TH BMD (R=−0.650, *p* = 0.058), respectively. A negative correlation was also demonstrated between the RQ value of *SFRP1* and LS BMD (R=−0.667, *p* = 0.049) and LS T-score (R=−0.835, *p* = 0.005). Table 2 presents the obtained correlation values between the RELs of studied genes and BMD and the number of recorded fracture events in the whole population, as well as in particular groups.

**TABLE 2:**
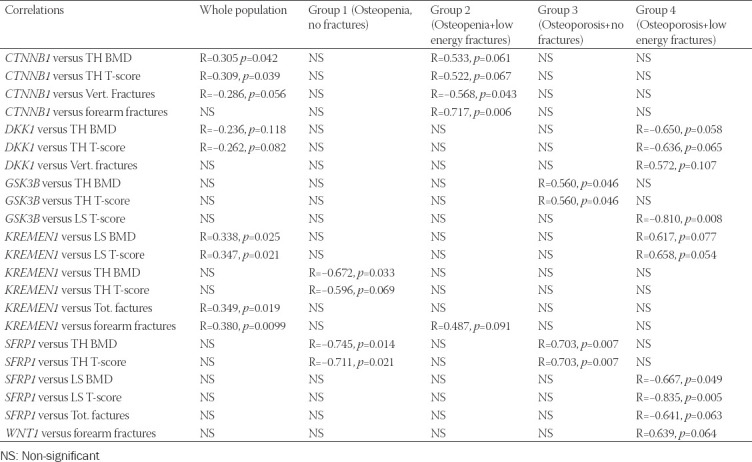
Correlations between gene RELs and either BMD or fractures

### Gene expression versus fractures

A negative correlation level on the borderline of statistical significance was found in the whole study population between the REL of *CTNNB1* and vertebral fractures (R=−0.286, *p* = 0.056). Positive correlations between the expression of *KREMEN1* and the total number of fractures (R=0.349, *p* = 0.019) and the number of forearm fractures (R=0.380, *p* = 0.0099) were also observed.

Moreover, we found out a correlation between the *CTNNB1* expression and the incidence of forearm and spinal fractures (R=0.717, *p* = 0.006 and R=−0.568, *p* = 0.043, respectively) in the subpopulation of patients with osteopenia and fractures (Group 2).

Patients in the group with advanced osteoporosis (Group 4) demonstrated a correlation on the borderline of statistical significance between: the number of spine fractures and *DKK1* gene expression (R=0.572 *p* = 0.107) and the total number of all fractures and the expression level of *SFRP1* (R=−0.641, *p* = 0.063) and the number of forearm fractures and *WNT1* expression (R=0.639, *p* = 0.064). Table 2 illustrates the results of correlation between RQ values of the studied genes versus the number of recorded fractures.

### Gene expression versus laboratory test results

We have observed many interesting correlations between the laboratory results and studied gene expression levels. Table 3 illustrates the results of correlation between RQ values of the studied genes and the results of laboratory tests.

**TABLE 3:**
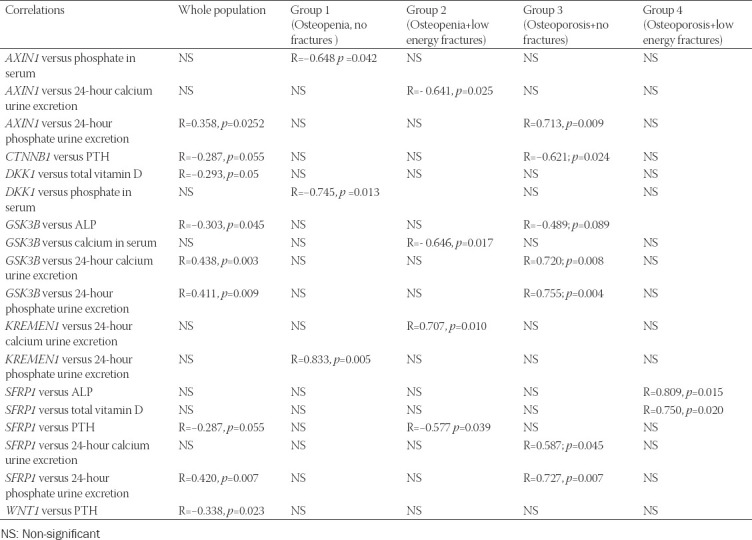
Correlations between gene expression levels and laboratory test results

## DISCUSSION

In the entire population, we observed a distinct overexpression of *SFRP1* in 100% of the study participants. In addition, the RELs of *DKK1, APC*, and *GSK3B* were slightly ­elevated, while the expression of the *KREMEN1* was similar to comparator. In contrast, RELs of *CTNNB1* and *AXIN1* were slightly decreased.

Roforth et al. [21] demonstrated the expression of *SFRP1* to be 1.6 higher in a bone material from elderly patients than in that from working-age subjects, which did coincide with our results. Reppe et al. [22] evaluated REL in a bone samples collected from healthy females with low or normal bone mass. Unlike in case of our results, the REL of *DKK1* was decreased in their whole population, while the expression of the *SFRP4* was increased. The use of HRT, reported by almost a half of the participants, could have been responsible for the differences in *DKK1* expression versus our results. Bolamperti et al. [23] compared Wnt pathway gene expression in bone samples, collected from patients with osteoporosis and/or osteoarthrosis (OA) that were qualified to hip arthroplasty. Similarly, as in our investigation, the expression of *CTNNB1* was in both groups decreased, however, differently than in our experiment, the *DKK1* expression was comparable to the reference gene. Another team also obtained analogical results of *CTNNB1* expression [24].

In our study, the lowest REL was observed for *CTNNB1* and its level decreased inversely to the age of patients with advanced osteoporosis. The decrease of *CTNNB1* expression may be a consequence of age-related suppression of the Wnt system, which is not always followed by a decrease in its serum concentration [25-27]. It should, however, be kept in mind that the expression of β-catenin increases in other diseases, among others in OA [28, 29].

We observed many significant correlations between the REL of the studied genes and DXA parameters or fractures. The studies evaluating the relationship between BMD and the Wnt pathway gene expression are rather scarce. It is probably due to the difficulties with obtaining material.

Reppe et al. [22], differently than in our study, demonstrated the presence of positive correlations between BMD, T-Score and Z-score of the hip and LS and the *DKK1* and *SOST* expression. The differences in selection of study population may be a possible explanation of the differences in results. The strongest correlations in our study were present between TH T-score and BMD and *DKK1* gene expression in the group with advanced osteoporosis.

In the study of Bolamperti et al. [23], both groups had comparable BMD. The expression of the WNT pathway activators: *WNT3* and *WNT10B*, was comparable in both groups, similarly as the *CTNNB1*, which was consistent with our results. In turn, the expression of Wnt signaling inhibitors, such as: *SOST*, *SFRP2*, and *DKK1*, was significantly lower in the group of patients after hip fractures. D`Amelio et al. [30] used the research model as above mentioned. The authors observed that patients after fracture had higher expression of *RANKL, M-CSF, SOST*, and, in contrast to our results, also of the *DKK1*. In our other study, we demonstrated that *RANK* and *RANKL* expression, correlated with changes of the hip region and LS BMD, while the type of applied therapy was significantly relevant as well [31]. Velasco et al. [29] showed differences in the expression of 55 genes related to the Wnt pathway, between groups of patients: With the OA of the hip, OA of the spine and those with osteoporosis. In most cases, the expression of the studied genes, including *CTNNB1* and *WNTB2*, was higher in patients with osteoarthritis compared to the group with fractures [29]. In the entire population of our study, we observed a distinct overexpression of the *WNT1*, which encodes another activator of the Wnt pathway.

Analogous results of the *CTNNB1* were obtained by another team [24]. However, the researchers demonstrated higher concentration of the β-catenin in the group without fractures, thus, most apparently, a post-translational suppression of the Wnt pathway activity could have occurred.

The obtained results of the correlations between the gene expression and laboratory results seem to be rather divergent. Some of the obtained results appear to be contrary to literature data, for example, the reverse correlation between the expression of *CTNNB1* and PTH concentration. It is known that PTH inactivates *GSK3B* protein and is responsible for Dvl connection to the PTHR, which is stabilizing β-catenin. In addition, PTH reduces the expression of the Wnt pathway inhibitors: Sclerostin and Dkk1 in osteocytes [15,32,33].

We are aware of limitations of our study. We studied REL in PBMC – an easily available material, which certainly cannot fully reflect the bone cells. Another limitation was a small number of patients. One should also remember that Wnt pathway is regulated by other receptors and their ligands, for example, bone morphogenic proteins, TNFα, TGFβ, PTH, and correlated signaling paths, for example, OPG/RANK/RANKL system [15,32-34]. Epigenetic control mechanisms may have also contributed to the lack of expected differences in gene expression [35,36]. Additionally, in the majority of studies material was collected from unhealthy people (e.g., the necessity for arthroplasty) and it was not possible to compare the obtained samples with material from healthy patients.

The Wnt pathway activity in osteoporosis seems to be suppressed, what leads to a decreased expression of the β-catenin-dependent genes. In OA, we observed activation of the Wnt pathway, what enhances the synthesis of matrix metalloproteinases, causing cartilage degradation, but may also induce a local anabolic effect [37-41].

We assume that the applied research model seems to be fairly promising; however, it is currently useful mainly in the context of comparison with parallel studies on bone material.

## CONCLUSION

In our opinion, the obtained research results allow us to conclude that high expression level of β-catenin (CTNNB1) ensures the maintenance of normal BMD and/or protects against fractures. Moreover, the changes in the expression levels of Wnt pathway genes in PBMC seem to reflect the expected changes in bone tissue.

The reliability and possibility of applied research model applications require further studies on larger groups and a comparative reference to bone material.

## Figures and Tables

**SUPPLEMENTARY TABLE 1:**
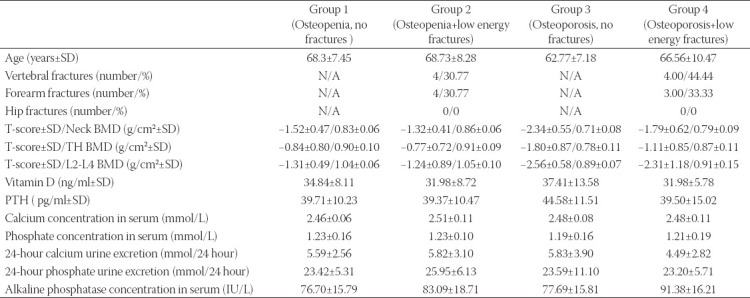
Baseline characteristics of the study patients

**SUPPLEMENTARY TABLE 2:**
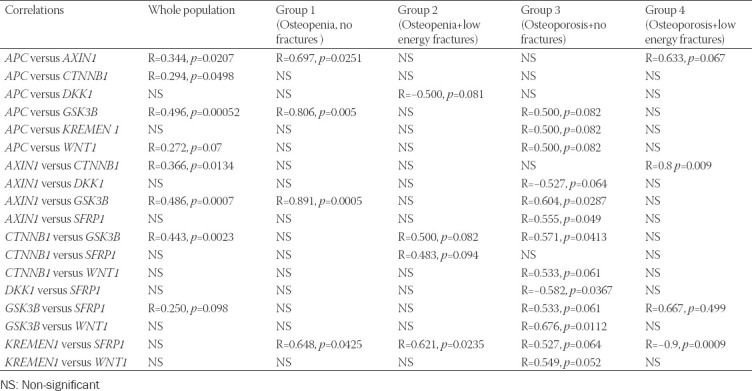
The results of correlations between RQ values, both in the whole study population and in subgroups (1-4)
